# Plasma Membrane Localization of CD36 Requires Vimentin Phosphorylation; A Mechanism by Which Macrophage Vimentin Promotes Atherosclerosis

**DOI:** 10.3389/fcvm.2022.792717

**Published:** 2022-05-18

**Authors:** Seo Yeon Kim, Se-Jin Jeong, Ji-Hae Park, Wonkyoung Cho, Young-Ho Ahn, Youn-Hee Choi, Goo Taeg Oh, Roy L. Silverstein, Young Mi Park

**Affiliations:** ^1^Department of Molecular Medicine, College of Medicine, Ewha Womans University, Seoul, South Korea; ^2^Department of Life Sciences, Immune and Vascular Cell Network Research Center, National Creative Initiatives, Ewha Womans University, Seoul, South Korea; ^3^Department of Physiology, College of Medicine, Ewha Womans University, Seoul, South Korea; ^4^Department of Medicine, Medical College of Wisconsin, Milwaukee, WI, United States

**Keywords:** vimentin, atherosclerosis, CD36, macrophage, intracellular trafficking

## Abstract

Vimentin is a type III intermediate filament protein expressed in cells of mesenchymal origin. Vimentin has been thought to function mainly as a structural protein and roles of vimentin in other cellular processes have not been extensively studied. Our current study aims to reveal functions of vimentin in macrophage foam cell formation, the critical stage of atherosclerosis. We demonstrated that *vimentin* null (*Vim*^–/^*^–^*) mouse peritoneal macrophages take up less oxidized LDL (oxLDL) than *vimentin* wild type (*Vim*^+/+^) macrophages. Despite less uptake of oxLDL in *Vim*^–/^*^–^* macrophages, *Vim*^+/+^ and *Vim*^–/^*^–^* macrophages did not show difference in expression of CD36 known to mediate oxLDL uptake. However, CD36 localized in plasma membrane was 50% less in *Vim*^–/^*^–^* macrophages than in *Vim*^+/+^ macrophages. OxLDL/CD36 interaction induced protein kinase A (PKA)-mediated vimentin (Ser72) phosphorylation. *Cd36*^–/^*^–^* macrophages did not exhibit vimentin phosphorylation (Ser72) in response to oxLDL. Experiments using phospho-mimetic mutation of vimentin revealed that macrophages with aspartate-substituted vimentin (V72D) showed more oxLDL uptake and membrane CD36. *LDL receptor* null (*Ldlr*^–/^*^–^*) mice reconstituted with *Vim*^–/^*^–^* bone marrow fed a western diet for 15 weeks showed 43% less atherosclerotic lesion formation than *Ldlr*^–/^*^–^* mice with *Vim*^+/+^ bone marrow. In addition, *Apoe*^–/^*^–^Vim^–^*^/^*^–^* (double null) mice fed a western diet for 15 weeks also showed 57% less atherosclerotic lesion formation than *Apoe*^–/^*^–^* and *Vim*^+/+^mice. We concluded that oxLDL via CD36 induces PKA-mediated phosphorylation of vimentin (Ser72) and phosphorylated vimentin (Ser72) directs CD36 trafficking to plasma membrane in macrophages. This study reveals a function of vimentin in CD36 trafficking and macrophage foam cell formation and may guide to establish a new strategy for the treatment of atherosclerosis.

## Introduction

CD36 is an 88 kDa plasma membrane glycoprotein and one of the major scavenger receptors expressed in various cell types including macrophages, microvascular endothelial cells and adipocytes. CD36 involves in many biological activities through binding to variety of ligands including modified low density lipoprotein (LDL), lipopolysaccharides of bacterial cell wall, and thrombospondin-1. In particular, CD36 binding to oxidized LDL (oxLDL) mediates uptake of oxLDL and leads to macrophage foam cell formation, the initial critical stage of atherosclerosis (1). *Ex vivo* experiments demonstrate that 60–70% of macrophage foam cell formation is induced by CD36-mediated oxLDL uptake (2). Although foam cell formation is a critical stage of atherosclerosis, the molecular mechanism by which macrophages uptake oxLDL has not been clearly defined. OxLDL/CD36 interaction provokes signals through activation of Lyn/MAPKK4/JNK2 in macrophages (3). Several studies elucidate that CD36-mediated oxLDL uptake mechanism is independent of caveloae, clathrin, and actin cytoskeleton but dependent on dynamin (4). It has been reported that the C-terminal six amino acids of the CD36 cytoplasmic tail are critical for binding and endocytosis of OxLDL (5). Therefore, elucidating the mechanism of oxLDL uptake via CD36 is warranted.

Vimentin is a 55 kDa protein, composing the major type III intermediate filament in cells of mesenchymal origin such as macrophages and adipocytes (6). Vimentin composes cytoskeletal networks from nuclear periphery to the cell membrane and functions in distribution of cellular organelles (7), cell migration and cell adhesion (8, 9). In clinical medicine, vimentin is commonly used for a marker for epithelial to mesenchymal transition (EMT) of cancer cells (10). Recent studies reported that vimentin is a component of lipid droplets in adipocytes (11) and influences lipid stability during adipocyte differentiation (12). However, the mechanism has not been fully elucidated. Vimentin plays a role in endocytosis of certain virus (13) and metal ion in fibroblast (14). Nevertheless, there have been few studies for the role of vimentin in functions other than cellular structure maintenance.

Vimentin has a highly conserved alpha helix domain and is capped on each end by amino-, carboxyl domain (15, 16). Two vimentin monomers form a coiled-coil structure and associate other homodimer to produce soluble tetramer that is a longitudinal unit of vimentin filament (17). Vimentin phosphorylation regulates the structure of vimentin, inducing formation of intermediate filaments or disassembly into vimentin monomers. It also changes affinity of vimentin to its binding partners. Vimentin has phosphorylation sites that are modulated by 10 kinases including PKC, PKA, and Cdk1 (18, 19). Phosphorylation-mediated regulation of vimentin has been well studied in cytokinesis. Cdk1 phosphorylates Ser55 on vimentin, leading to depolymerization of vimentin filament from prometaphase to metaphase (20, 21). Ser38 and Ser72 on vimentin are phosphorylated by PKA and lead to disassembly of vimentin filaments in fibroblast. However, the function of phospho-vimentin (Ser72) in macrophages has not been studied.

Atherosclerosis is an important underlying pathology of cardiovascular disease, which is currently the leading cause of mortality worldwide (22). Therefore, verifying the mechanism of macrophage foam cell formation should guide to develop a new strategy for the treatment of atherosclerosis (23–28).

A recently published paper written by Haversen et al. showed that loss of vimentin increased macrophage surface CD36 expression *in vitro*, however, reduced atherosclerosis in two animal models including LDL receptor null (*LDLR*^–/–^) mice reconstituted with vimentin null (*Vim*^–/–^) bone marrow and *Vim*^–/–^ mouse injected with PCSK9 gain-of-function virus (29).

In the current study, we performed experiments to evaluate how vimentin deficiency affects macrophage foam cell formation and atherosclerosis. Our current study reveals that vimentin plays a role in CD36 trafficking to plasma membrane and contributes to macrophage foam cell formation. In addition to *in vitro* experiments, our *in vivo* experiments using *vimentin* null (*Vim*^–/–^) bone marrow transplantation into hypercholesterolemic mice confirmed the atherosclerosis-promoting effect of vimentin. Regarding the functions of CD36 in various biological activities including immunity and anti-angiogenesis, the mechanism of CD36 trafficking may guide ways to modulate the cellular processes mediated by CD36.

## Materials and Methods

### Regent

Oil red O (ORO), 1,10-dioctadecyl-3,3,30,30-tetramethylindocarbocyanine perchlorate (DiI) and human AB serum were obtained from Sigma (Sigma, United States). The intracellular cholesterol assay kit was from Cayman (Cayman Chemical Co, United States). Antibodies for CD36, Vimentin, tubulin, EEA1 and beta-actin were obtained from Abcam (Abcam, United States). Antibodies for SRA1 conjugated with APC and control antibody were manufactured by Bioyrt (Bioryt, United States). Antibodies for CD36 conjugated with APC and suitable control antibody were from Bio-Rad (Bio-Rad, United States). Sucrose, dextrose, SDS were obtained from Ducefa (Ducefa, Germany). Antibodies for rabbit IgG, IgM were purchased from bethyl chemisty (bethyl, United States). Antibody for p-PKAα/β/γ cat (Thr 198) was from Santa Cruz Biotechnology (sc-32968) (Santa Cruz, United States).

### Mouse Protocol

Pathogen-free, male C57BL/6 mice, 6–8 week old were purchased from Orient Bio (Orient Bio, South Korea). *Vimentin* null (*Vim* ^–/–)^ mice on a 129 background were provided by Dr. John E. Eriksson (Åbo Akademi University, Finland) and seven times backcrossed to C57Bl/6. *LDL receptor* null (*LDLr* ^–/–^) mice were provided by Dr. Goo Taeg Oh (Ewha Womans University, Seoul, South Korea). In all experiments, age-matched (7–10 weeks old) male mice were used. The Institutional Animal Care and Use Committee (IACUC) of Ewha Womans University College of Medicine approved the experimental protocol (IACUC approval No. ESM-12-0198).

### Bone Marrow Transplantation and Atherosclerosis Analysis

*LDLr*^−/–^mice were reconstituted with bone marrow from *Vim*^+/+^ mice (*n* = 11) and *Vim*^−/–^ mice (*n* = 10). To induce bone marrow aplasia, *LDLr*^−/–^mice (male, age 6 weeks) were exposed to a single dose of 13 Gy (0.28 Gy/min, 200 kV, 4 mA) x-ray (total body irradiation) with a 4 mm aluminum filter, 1 day before the transplantation. Bone marrow cell suspensions were isolated by flushing the femurs and tibias from either male *Vim*^+/+^ or *Vim*^–/–^ mice with phosphate-buffered saline. Irradiated recipients received 1.5 × 10^7^ bone marrow cells by intravenous injection into the tail vein. After 4 weeks, reconstituted *Ldlr*^−/–^ mice were fed a western diet for 15 weeks.

We generated *Apoe***^–^**^/^**^–^** *Vim* **^–^**^/^**^–^** (double null) mice by crossing the *Apoe***^–^**^/^**^–^** and *Vim* **^–^**^/^**^–^** mouse strains. *Apoe***^–^**^/^**^–^** *Vim* **^–^**^/^**^–^** mice and *Apoe***^–^**^/^**^–^** *Vim*^+/+^ mice were fed a western diet for 15 weeks.

Western diet-fed mice were euthanized and perfused with cold 0.01 mol/L PBS through left ventricle. Aortae were dissected from the proximal ascending aorta to the bifurcation of iliac artery and fixed in 4% buffered paraformaldehyde for 24 h. After fixation, the aortae were split longitudinally and pinned open for surface lesion measurements with 0.5% Oil-Red O staining. For aortic sinus analysis, heart was embedded in optimal cutting temperature compound (OCT), and snap-frozen in liquid nitrogen. Serial 7-μm cryosections of the aortic sinus were cut using a Leica CM1950 cryostat. Four or more frozen section slides for aortic sinus were made per mouse. Cryosections were fixed in 4% buffered paraformaldehyde and stained in 0.5% Oil Red O for 25 min. Quantitative analysis of atherosclerotic lesions was performed using the Axiovision release 4.4 software (Carl Zeiss, Germany) program.

### Low Density Lipoprotein Preparation and Oxidation

LDL was obtained from human plasma via density gradient ultracentrifugation (30). Oxidized LDL (oxLDL) was generated by dialysis of LDL with 5μM CuSO_4_ in PBS for 6 h at 37^°^C. To terminate oxidation of LDL, LDL was dialyzed with 100 μM EDTA in PBS.

**FIGURE 1:**
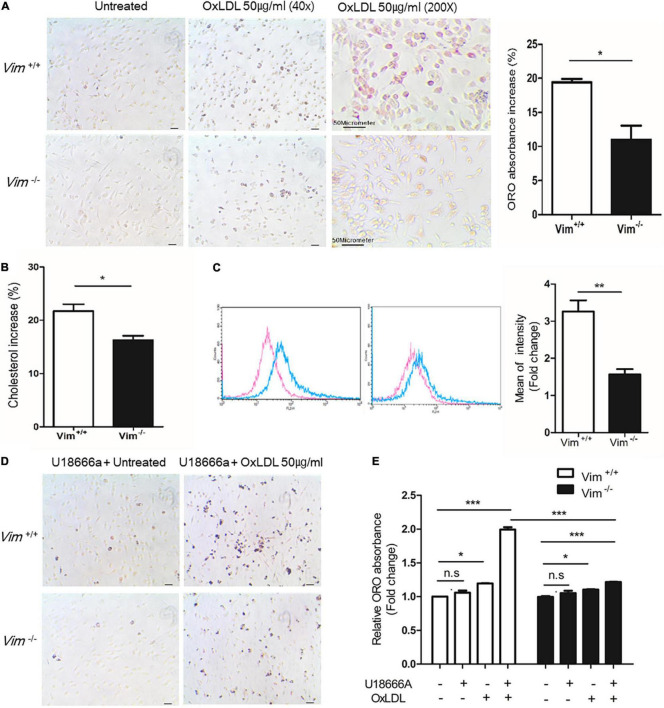
*Vim*^−/–^ murine peritoneal macrophages exhibit less foam cell formation and less uptake of oxLDL than *Vim*^+/+^ macrophages. **(A)** Left, Representative images of ORO staining of *Vim*^+/+^ mouse macrophages and *Vim*^−/–^ mouse macrophages. Both macrophages were incubated with or without oxLDL (50 μg/ml) for 18 h. Right: the absorbance of ORO eluted from macrophages in **(A)**. The absorbance increases induced by oxLDL treatment were compared between *Vim*^+/+^ and *Vim*^−/–^ macrophages. **(B)** Increases of Intracellular cholesterol concentration in *Vim*^+/+^ mouse macrophages and *Vim*^−/–^ mouse macrophages were compared after incubation with oxLDL 50 μg/ml for 18 h. **(C)** Left, Flow cytometry. *Vim*^+/+^ mouse macrophages and *Vim*^−/–^ mouse macrophages were pretreated with non-labeled oxLDL for 30 min, and then exposed to oxLDL complexed with DiI (DiI-oxLDL, 50 μg/ml) for 5 min and fixed with 4% paraformaldehyde. The fluorescence intensities of these cells were measured using flow cytometry. Red line; without oxLDL, Blue line; with oxLDL. Right graph: Geometric mean fluorescence intensities were compared between *Vim*^+/+^ and *Vim*^−/–^ macrophages incubated with oxLDL. The bar graph of comparison was generated from 3 times of separate experiments. **(D)** Representative images of ORO staining of *Vim*^+/+^ and *Vim*^−/–^ mouse macrophages. Both macrophages were pre-incubated with U18666a (NPC inhibitor, 10 nM) for 90 min and then incubated with or without oxLDL (50 μg/ml) for 18 h. **(E)** Relative ratio of absorbance of eluted ORO in **(A,D)**. The ORO was extracted using isopropanol and the absorbance representing intracellular lipids was detected at 540 nm. (*p < 0.05, **p < 0.01, ***p < 0.001. The graph shows mean ± SEM for triplicated determinants of 3 separate experiments).

In addition to CuSO_4_-oxLDL, we generated oxLDL modified by myeloperoxidase (MPO) and used in several experiments as Figure 1D. LDL prepared from human plasma by density gradient ultracentrifugation was oxidatively modified by incubation in a buffer containing 50 mM sodium phosphate (pH 7.0) and 100 μM diethylene triamine pentaacetate (DTPA) with 30 nM MPO, 100 μg of glucose, glucose oxidase at 20 ng/ml (grade II; Boehringer Mannheim Biochemicals, Penzberg, Germany), and 0.5 mM NaNO_2_ at 37^°^C for 8 h (oxLDL) (31). The oxidation reaction was terminated by the addition of 40 μM butylated hydroxyl-toluene and 300 nM catalase to the reaction mixture.

### Cell Culture

Peritoneal macrophages were collected by peritoneal lavage of mice 4 days after intraperitoneal injection of 4% thioglycolate (1 ml). Mice were euthanized with CO_2_ before harvesting macrophages. Cells were cultured in RPMI containing 10% fetal bovine serum and 1% penicillin-streptomycin. Media was changed to serum-free RPMI for treatment with oxLDL or other chemicals.

Primary bone marrow-derived macrophages (BMDMs) were collected from *Vim* ^–/–^ mice and wild type mice as described previously (32). Briefly, BMDMs were differentiated from murine bone marrow myeloid stem cells and cultured for 7 days with DMEM supplemented with 10% L929 supernatant containing 10% fetal bovine serum (FBS), 1 mM sodium pyruvate, 1% penicillin-streptomycin and 5 × 10–5M 2-mercaptoethanol at 37^°^C and 5% CO_2_.

SW13 cells were purchased from ATCC (ATCC, United States) and incubated following their protocol.

### Oil Red O Staining

ORO staining was performed using murine peritoneal macrophages plated in 12-well plate as described previously (33). Cells were incubated with or without 50μg/ml of oxLDL for 18 h. Then macrophages were fixed in 4% paraformaldehyde in PBS for 20 min at room temperature. To stain the intracellular lipids, Cells were washed with PBS twice and treated with 60% isopropanol for 5 min and ORO solution (diluted 2:3 of H2O:0.3% ORO in isopropanol) for 15 min. After rinsing with distilled water, cells were examined microscopically.

We added 500 μl of isopropanol onto the ORO-stained macrophages to extract the ORO incorporated into the intracellular lipids. The amount of the ORO flowing out from the macrophages was measured by using spectrophotometry (absorbance at 510 nm).

### Western Blotting

Cells were lysed in buffer containing 1% triton X-100, 20 mM Tris-HCl (pH 7.5), 150 mM NaCl, 1 mM EDTA, 1 mM EGTA and protease inhibitor cocktail (Roche, Germany) and phosphatase inhibitors (10 mM phenylmethylsulfonyl fluoride/PMSF, 1% sodium pyrophosphate, 10 mM sodium fluoride, and 2 mM sodium vanadate). The lysates were separated by SDS-PAGE, transferred to PVDF membrane (Millipore), and analyzed by immunoblotting. Membranes were probed with antibodies for vimentin, CD36, phospho-vimentin (Ser72), and phospho-vimentin (Ser38). Antibodies for β-actin and GAPDH were used for normalization. Band intensities were quantified using Image J program (U. S. National Institutes of Health, United States).

### Flow Cytometry

Cells were incubated in 6 well plates with appropriate media containing 10% FBS. After rinsing twice with PBS, cells were harvested gently with scrapers and moved to round bottom glass tubes. After centrifuge at 1,000 × g in glass tubes, cells were incubated with 5% BSA for 30 min at 4^°^C and then incubated with APC-conjugated anti-CD36 antibody (Bio legend, United States), FITC-conjugated anti-MSR1 antibody or isotype control (Bio legend, United States) diluted in PBS containing 5% BSA for 1 h at 4^°^C. After three washes with PBS, cells were analyzed by flow cytometry.

### Immunoprecipitation

Cells were lysed with RIPA buffer (1% triton x-100, 0.1% sodium dodecyl sulfate, 0.5% deoxycholate, 50 mM Tris-HCl (pH 7.5), 150 mM NaCl) and protease inhibitor cocktail. The protein concentrations of the lysates were quantified by BCA method and appreciate amounts of lysates were pre-incubated with Protein A or Protein A/G plus bead (Santa cruz, United States) for 2 h. The supernatant containing 350 mg protein was incubated with 2 μg of anti-CD36 antibody or anti-vimentin antibody overnight at 4^°^C. Protein A or Protein A/G plus beads were added to the lysates for 4 h. Beads were extensively washed, boiled in laemmli buffer (Bio-rad, United States) and the bead-bound material was analyzed by immunoblotting for CD36, vimentin or PKA.

### 1,10-Dioctadecyl-3,3,30,30-Tetramethylindocarbocyanine Perchlorate Oxidized LDL Uptake Assay

OxLDL was complexed with DiI as described previously. OxLDL (500 μg/ml) was mixed with 10 μg/ml of DiI in DMSO at 37^°^C for 16 h. DiI-OxLDL solution was dialyzed in PBS. Macrophages were cultured in 6 well plates and then incubated with oxLDL (50 μg/ml) at 37^°^C for 30 min. Cells were treated with DiI-oxLDL 50 μg/ml for 5 min, fixed in 4% paraformaldehyde in PBS and washed with PBS. Cells were collected by scrapping and spinned at 1,000 g for cell debris removal. DiI-OxLDL taken up by macrophages was measured using flow cytometry.

### Intracellular Cholesterol Concentration Measurement

Intracellular cholesterol was measured using a cholesterol fluorometric assay kit (Cayman). *Vim*^+/+^ macrophages and *Vim*^–/–^ macrophages were cultured in 6 well plates with or without oxLDL (50 μg/ml) and cells were washed twice with PBS after 18 h. Assay buffer including 0.5% triton X-100 was added onto the cells. The cell lysates were centrifuged at 13,000 × g for 30 min at 4^°^C. The supernatants were used for the cholesterol measurement. The value of intracellular cholesterol was normalized by comparison to the protein concentration of the sample.

### RNA Isolation and Real Time PCR

Total RNA was extracted from macrophages using Trizol reagent (Invitrogen, United States) according to the manufacturer’s instruction. We used iScript cDNA synthesis kit (Bio-rad, United States) to synthesize cDNA. Quantitative real-time reverse transcriptase PCR (qRT-PCR) was performed with Power SYBR Green PCR Master Mix (Applied Biosystems, United States) and an ABI Real-Time PCR thermocycler. RNAs were analyzed by qRT-PCR with following primers (5′-CCC AGA GCA AAA AGC GAC TC-3′ and 5′- GGT CAT CAT CAC TTT GGT CCT TG-3′ for ABCA1, 5′-CAA GAC CCT TTT GAA AGG GAT CTC-3′ and 5′-GCC AGA ATA TTC ATG AGT GTG GAC-3′ for ABCG1, 5′-GGC TGC TGT TTG CTG CG-3′ and 5′-GCT GCT TGA TGA GGG AGG G-3′ for SR-B1, 5′-GAT CGG AAC TGT GGG CTC AT-3’ and 5′-GGT TCC TTC TTC AAG GAC AAC TTC-3’ for CD36, 5′-AAA GAA GAA CAA GCG CAC GTG G-3’ and 5′-GAG CAC CAG GTG GAC CAG TTT G-3’ for SR-A1, and 5′-TCC ATG ACA ACT TTG GCA TTG-3’ and 5′- TCA CGC CAC AGC TTT CCA-3’ for GAPDH.) GAPDH was used for internal control.

### Vector Construction

All plasmids were produced using standard cloning techniques. Vimentin sequence was cloned into pAcGFP-Hyro-N1 vector for imaging. pLVX-puro vector were used for mutagenesis. These vectors were provided by Dr. Youngho Ahn (Ewha Womans University, Seoul, South Korea). Vimentin construct was amplified by PCR with following primers (VIM -HindIII-F: GTCA AA GCT TCG ATG TCC ACC AGG TCC, GTG TC, VIM -HindIII-R: GTCA AA GC TTA TTC AAG GTC ATC GTG ATG CTG, VIM -EcoRI-Flag-F: GTCA GAATTC GCCACC ATG GAT TAC AAG GAT GAC GAC GAT AAG ATG TCC ACC AGG TCC GTG TC, VIM -EcoRI-R: GTCA GAATTC TTA TTC AAG GTC ATC GTG ATG CTG) in reactions as follows; denaturation at 94^°^C for 5 min, followed by 30 cycles of denaturation at 94^°^C for 2 min, annealing at 59^°^C for 1 min, and extension at 72^°^C for 2 min. A final elongation step was carried out at 72^°^C for 10 min.

For site-directed mutagenesis, we used polymerase chain reaction (PCR) with oligonucleotide mutation primers and the template vimentin cDNA. The primers for site-directed mutagenesis are VIM -S72A-F: GCC GTG CGC CTG CGG GCC AGC GTG CCC GGG GTG, VIM -S72A-R: CAC CCC GGG CAC GCT GGC CCG CAG GCG CAC GGC, VIM -S72D-F: GCC GTG CGC CTG CGG GAC AGC GTG CCC GGG GTG, VIM -S72D-R: CAC CCC GGG CAC GCT GTC CCG CAG GCG CAC GGC. The mutation sites were confirmed by sequencing by Cosmo Genetech.

### Lentivirus-Mediated Transfection

Genetically modified lentiviruses were produced by transient transfection of 293T cells with Plvx -VIM, Plvx -V72A, Plvx -V72D, Plvx-puro, PAX2 packaging plasmid (containing gag and pol gene of HIV) and MDG2 envelop plasmid (containing vesicular stomatitis viral glycoprotein expressing vector) using Lipofectamine 2000 reagents (Thermo Fisher Scientific, United States).

A293T cells were seeded onto 6 well plates at 50∼70% confluence 1 day before the transfection. Transfection mixture containing 6 μg target vector, 5 μg PAX2, 3 μg MDG2, and 10 μg/ml Lipofectamine was added to the A293T cells. The media was collected after 24 h, filtered through a 45 μm pore filter and added to the recipient cells. To enhance the transfection efficiency, virus-containing media was mixed with polybrene 5 μg/ml (Millipore). We performed second transfection repeating the same procedure 24 h after the first transfection to promote viral incorporation.

### Immunocytochemistry and Immunohistochemistry

Cells grown on glass coverslips were fixed with 4% formaldehyde in ice-cold PBS for 20 min and then 0.1% triton X-100 in ice-cold PBS for 5 min. Cells were incubated with 5% BSA in PBS for 1 h to reduce non-specific signals and then incubated with primary antibodies diluted in PBS containing 5% BSA for 16 h at 4^°^C. After 3 washes with PBS, cells were incubated with appropriate fluorescent dye-conjugated secondary antibodies for 1 h and washed with PBS. Then nuclei were stained with DAPI-containing mounting solution (Vector laboratories, United States). Fluorescently stained cells were examined under a Zeiss confocal microscope and analyzed by Zeiss imaging processor.

Aortic sinus tissues were embedded in optimal cutting temperature compound (SAKURA Tissue-Tek, United States) for frozen section and snap frozen at –80^°^C. These frozen tissues were sectioned on a cryostat, transferred to slides and then dried to preserve morphology. Sections were dried for 5 min at room temperature and were fixed by pre-cold acetone for 15 min. The following process was equal to immunocytochemistry.

### Early-Endosome Fraction Assay

Mouse peritoneal macrophages were incubated with oxLDL (50 μg/ml) for 10 min. After the designated time point, cells were washed twice with RPMI followed by the application of 0.5 ml of homogenization buffer [250 mM sucrose, 1 mM EDTA, 1 mM phenylmethylsulfonyl fluoride (PMSF)], in which cells were gently detached using a cell scraper, lysed, and further processed for examination by a sucrose flotation assay. Specifically, after centrifugation (1,000 × g), the post-nuclear supernatant was collected and adjusted to a concentration of 25% sucrose and 1 mM EDTA in 1 ml total volume. In 1 ml increments, 2.4 ml of 45% sucrose was transferred to the bottom of a SW41Ti tube and successively overlaid with 5.2 ml of 35% sucrose, 3.9 ml of 25% sucrose, and 1 ml of post-nuclear supernatant in 25% sucrose. These fractions were further analyzed using endosomal markers, CD36 and vimentin.

### Thiobarbituric Acid Reactive Substance Assay

Lipid peroxidation of LDL was measured by Thiobarbituric Acid Reactive Substance (TBARS) formation. Briefly, LDL and oxLDL were incubated with ice cold 10% Trichloroacetic acid to precipitate protein for 15 min on ice. And centrifuge samples 2,200 × g for 15 min at 4^°^C. Place supernatant into new labeled tube and add equal volume of 0.67% (w/v) Thiobarbituric Acid (TBA). Incubate in a boiling water bath for 10 min and record absorbance at 532 nm.

### Statistical Analysis

Data are expressed as mean ± standard error of the mean (SEM). Student’s *t*-test was used for comparisons between two sample means. ANOVA test was used to compare three or more groups. We also used non-parametric Kruskal–Wallis test for post-comparison hoc test. A *p*-value less than 0.05 were considered statistically significant. All experiments were repeated at least 3 times independently and all the measurements were done three times for a set. Analyses were performed using GraphPad Prism Software (GraphPad Software).

## Results

### *Vim*^–/–^ Murine Peritoneal Macrophages Exhibit Less Uptake of Oxidized LDL Than *Vim*^+/+^ Macrophages

To determine if vimentin plays a role in macrophage foam cell formation, we performed ORO staining with *Vim*^+/+^ and *Vim*^−/–^ murine peritoneal macrophages incubated with oxLDL for 18 h (Figure 1A). We extracted ORO from the ORO-stained macrophages and measured the absorbance to quantify the amount of intracellular lipids. The ORO incorporated in the *Vim*^−/–^ macrophages was 10% less than that in the *Vim*^+/+^ macrophages (Figure 1A). In accordance, the increment of intracellular cholesterol after the oxLDL treatment which was measured by cholesterol oxidase assay was 6% less in *Vim*^−/–^ macrophages than in *Vim*^+/+^ macrophages (Figure 1B). These data indicate that vimentin deletion reduces macrophage foam cell formation.

Previous reports revealed that vimentin functions in uptake of virus and metal ion such as zinc (13, 14, 34). To evaluate roles of vimentin in lipid uptake, peritoneal macrophages from *Vim*^−/–^ and *Vim*^+/+^ mice were treated with oxLDL complexed with diI (DiI-oxLDL) for 5 min and fixed with 4% paraformaldehyde. Our data from flow cytometry showed that the mean fluorescence intensity of macrophages representing DiI-oxLDL uptake was 50% less in *Vim*^−/–^ macrophages than in *Vim*^+/+^ macrophages (Figure 1C).

To clarify in which stage of oxLDL-internalization vimentin functions, we treated macrophages with U18666a (10 nM), Niemann-Pick C1 (NPC-1) inhibitor, to block the later process of endocytosis, especially the transport of LDL-derived cholesterol from lysosomes to endoplasmic reticulum (35). *Vim*^+/+^ and *Vim*^−/–^ mouse peritoneal macrophages were pre-incubated with U18666a (10 nM) for 90 min, incubated with or without oxLDL (50 μg/ml) for 18 h and stained with ORO (Figure 1D). The absorbance of ORO eluted from *Vim*^−/–^ macrophages was 1.3 fold increased after the oxLDL treatment while *Vim*^+/+^ macrophages showed 2.0 fold-increase after the oxLDL treatment (Figure 1E). The result suggested that less intracellular lipids in *Vim*^−/–^ macrophages were not caused by increased trafficking of free cholesterol from late endosome/lysosomes to other cellular compartments, but is caused by diminished uptake of lipoproteins.

### Plasma Membrane CD36 Was Less in *Vim*^−/–^ Macrophages Than in *Vim*^+/+^ Macrophages

**FIGURE 2:**
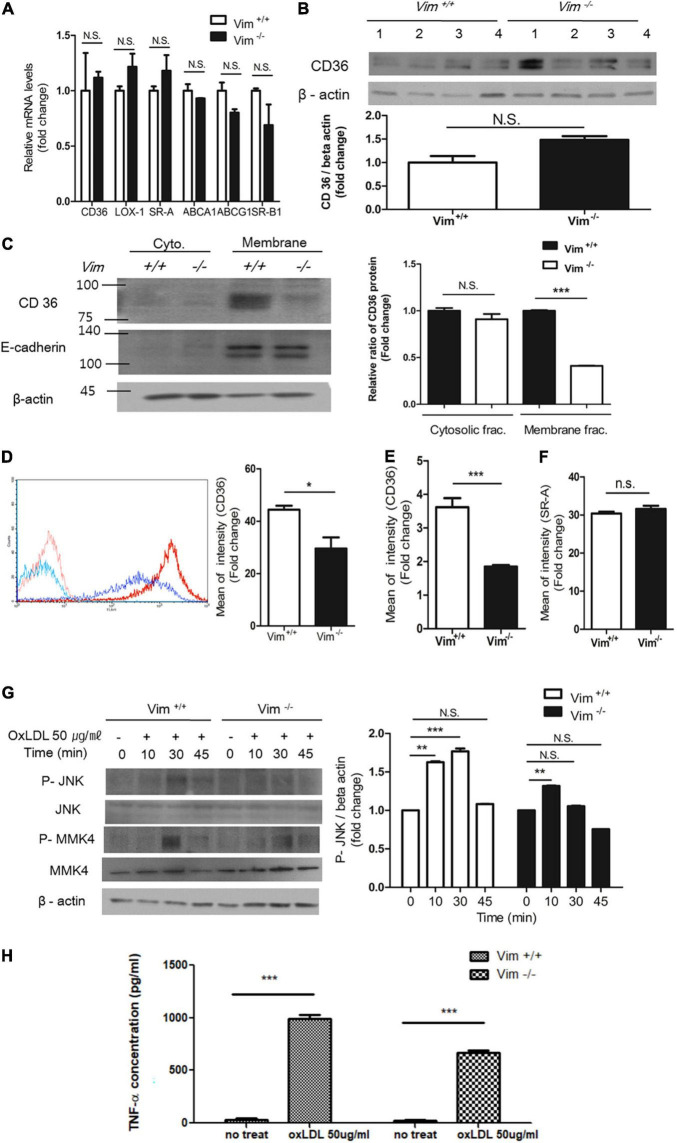
Plasma membrane CD36 in *Vim*^−/–^ macrophages was less than in *Vim* ^+/+^ macrophages. **(A)** Quantitative real-time PCR was performed with RNAs from *Vim* ^+/+^ and *Vim*^−/–^ murine peritoneal macrophages. GAPDH was used as the internal control. **(B)** Western blot analysis for CD36 was performed using lysates of *Vim* ^+/+^ (*n* = 4) and *Vim*^−/–^ (*n* = 4) murine peritoneal macrophages. Each lysates were harvested from different mice. Beta actin was used as the internal control. **(C)** Western blot analysis for CD36 was performed using fractionated lysates of *Vim* ^+/+^ and *Vim*^−/–^ murine peritoneal macrophages. E-cadherin was used as the marker for plasma membrane fraction and GAPDH was used as the marker for cytosolic fraction. **(D)** Left, The representative flow cytometry data of *Vim* ^+/+^ murine peritoneal macrophages and *Vim*^−/–^ murine peritoneal macrophages with APC conjugated monoclonal antibody specific for CD36. Both cell types were incubated with either IgG control (*Vim* ^+/+^: pink, *Vim*^−/–^: light blue) or with CD36 antibody (*Vim* ^+/+^: red, *Vim*^−/–^: dark blue). Right, Comparison of fluorescence intensities representing cell surface-localized CD36 between *Vim* ^+/+^ and *Vim*^−/–^ murine peritoneal macrophages. **(E)** Flow cytometry. Comparison of fluorescence intensities representing cell surface-localized CD36 between *Vim* ^+/+^ and *Vim*^−/–^ murine bone marrow-derived macrophages (BMDM). **(F)** Flow cytometry. Comparison of fluorescence intensities representing cell surface-localized SR-A between *Vim* ^+/+^ and *Vim*^−/–^ murine peritoneal macrophages. **(G)** Western blot analysis for phospho-JNK, phospho-MMK4 and beta actin using lysates of *Vim* ^+/+^ and *Vim*^−/–^ murine peritoneal macrophages. Cells were incubated with oxLDL (50 μg/ml) for indicated times. Beta actin was used as the internal control. *p < 0.05, **p < 0.01, ***p < 0.001. The graph shows mean ± SEM for triplicated determinants of the experiments. **(H)** *Vim*^+/+^ and *Vim*^−/–^ macrophages were treated with or without oxLDL (50 μg/ml) and TNF-α released by the macrophages in the media was measured by ELISA. ***p < 0.001.

Macrophages express various receptors including CD36 and scavenger receptor-A (SR-A) to uptake oxLDL. ABCA1 and ABCG1 mediate cholesterol efflux to apolipoprotein A1 and high density lipoprotein (36–38). Dysregulation of uptake and efflux of lipids leads to foam cell formation. We performed western blot analyses and quantitative real time PCR (RT-PCR) for receptors known to regulate cholesterol influx and efflux. Quantitative RT-PCR results showed no significant difference in expressions of CD36, LOX-1, SR-A, ABCA1, ABCG1, and SR-B1 between *Vim*^+/+^ and *Vim*^−/–^ mouse macrophages (Figure 2A). The western blot showed that CD36 protein levels were not different between *Vim*^+/+^ and *Vim*^−/–^ macrophages (Figure 2B).

However, plasma membrane-localized CD36 was significantly different between *Vim*^+/+^ and *Vim*^−/–^ macrophages. Subcellular fractionation and western blots using the cytoplasmic and membrane fractions of macrophage lysates revealed that plasma membrane-localized CD36 in *Vim*^−/–^ mouse peritoneal macrophages was 60% less than in *Vim*^+/+^ mouse peritoneal macrophages (Figure 2C). In accordance, flow cytometry showed that *Vim*^−/–^ macrophages exhibited less cell surface-localized CD36 than *Vim*^+/+^ mouse peritoneal macrophages (Figure 2D). Bone marrow-derived macrophages (BMDM) from *Vim*^−/–^ mice also showed 50% less cell surface-localized CD36 than BMDM from *Vim*^+/+^ mice in our flow cytometry data (Figure 2E). However, cell surface-localized SR-A measured by flow cytometry did not show difference between *Vim*^+/+^ and *Vim*^−/–^ mouse peritoneal macrophages (Figure 2F).

OxLDL via CD36 activates c-Jun N-terminal kinase (JNK) 1/2 through MKK4 in macrophages (3). OxLDL treatment induced phosphorylation of MKK4 and JNK 1/2 in *Vim*^+/+^ macrophages, however, *Vim*^−/–^ macrophages showed diminished responses to oxLDL (Figure 2G).

It has been reported that OxLDL/CD36 interaction induces release of cytokines including TNF-α (39). We measured TNF-α production in response to oxLDL in *Vim*^+/+^ and *Vim* **^–^**^/^**^–^** macrophages. *Vim*^+/+^ macrophage secreted 1.47 fold more TNF-α than *Vim* **^–^**^/^**^–^** macrophages (Figure 2H).

The results revealed that vimentin deletion decreases oxLDL/CD36-provoked signaling via less CD36 trafficking to plasma membrane.

### Oxidized LDL Induces Vimentin–CD36 Colocalization in Macrophages

**FIGURE 3:**
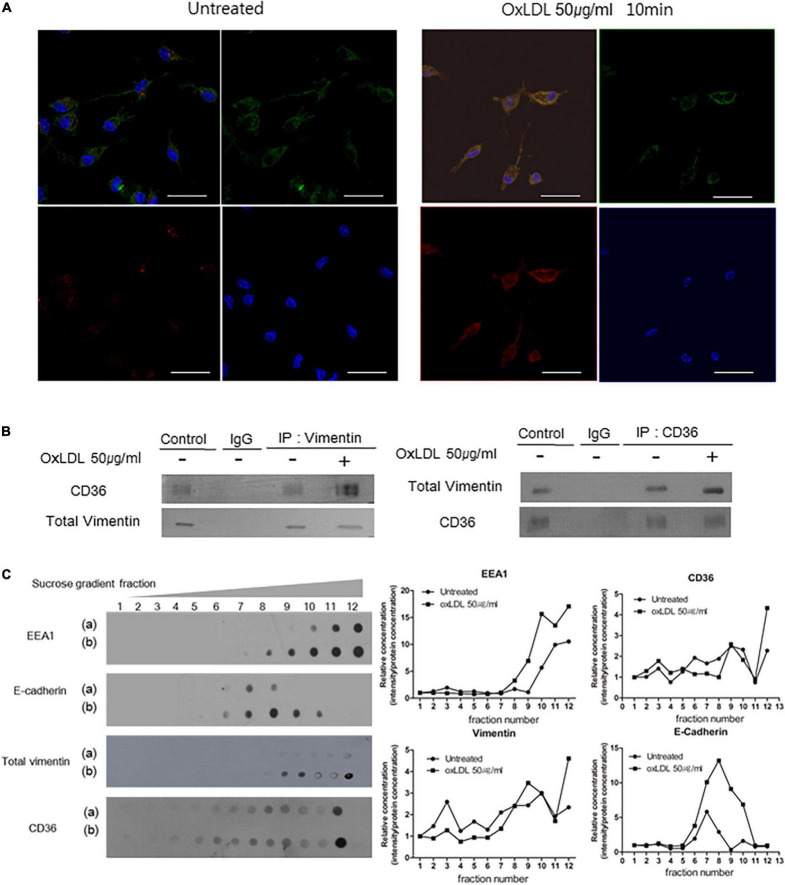
OxLDL induces CD36-Vimentin colocalization in early endosome. **(A)** Representative immunocytochemistry images of wild type macrophages incubated with or without oxLDL (50 μg/ml) for 10 min. Cells were stained with a rabbit polyclonal antibody to CD36 (Dylight 594; Red) and a mouse monoclonal antibody to vimentin (Alexa 488; Green). Scale Bars represent 20 μm. **(B)** *Vim* ^+/+^ mouse peritoneal macrophages were treated with or without oxLDL (50 μg/ml) for 10 min and immunoprecipitated with indicated antibodies. The precipitants were analyzed by western blot with antibodies against vimentin and CD36. **(C)** Sucrose gradient early endosome fraction test. Wild type macrophages were incubated with or without oxLDL (50 μg/ml) for 10 min and homogenized. The lysates were added to 15∼40% sucrose gradient column and centrifuged. (a) oxLDL-untreated and (b) oxLDL-treated macrophage lysates. Immunoblot analyses for indicated proteins.

Since we have found decreases in oxLDL uptake and CD36 trafficking that are attributed to vimentin deficiency, we tested if vimentin and CD36 are physically associated. The immunostaining showed that CD36 and vimentin are co-localized. The co-localization of vimentin and CD36, especially near the plasma membrane, was increased within 10 min after the oxLDL treatment compared with oxLDL-untreated macrophages (Figure 3A). Moreover, immunoprecipitaion assay results indicated that oxLDL increased association between vimentin and CD36 (Figure 3B).

We isolated endosomal fractions using sucrose gradient fractionation. Early endosomes were labeled by early endosome marker EEA1 and are in the 10th, 11th, and 12th fractions. There were CD36 and vimentin in early endosome fractions. In addition, the amounts of CD36 and vimentin in the early endosome fractions were increased when the cells were treated with oxLDL (50 μg/ml) (Figure 3C).

Overall, these results reveal that oxLDL promotes colocalization of CD36 and vimentin.

### Oxidized LDL/CD36 Interaction Induces Vimentin Phosphorylation at Serine 72 and Induces Disassembly of Vimentin Filaments

**FIGURE 4:**
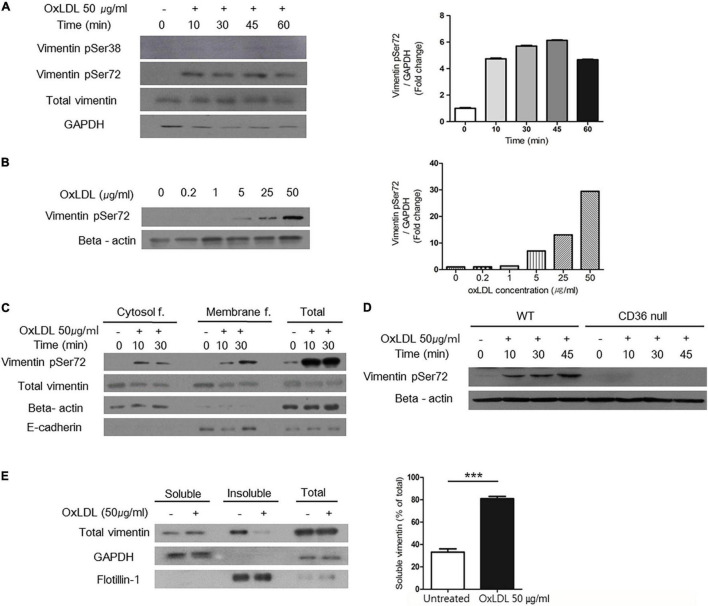
OxLDL-induced vimentin (Ser72) phosphorylation depended on CD36 and led to disassembly of vimentin filaments. **(A)** Western blots for phosphorylated vimentin (Ser72) and phosphorylated vimentin (Ser38) using cell lysates of wild type murine peritoneal macrophages. Cells were treated with oxLDL (50 μg/ml) for indicated times. **(B)** Western blot for phosphorylated vimentin (Ser72) using cell lysates of wild type murine peritoneal macrophages. Cells were treated with various concentrations of oxLDL for 10 min. **(C)** Wild type murine peritoneal macrophages were incubated with OxLDL (50 μg/ml) for indicated times. Cytosolic and membrane fractions were separated using buffer-based protocol. E-cadherin was used as a marker for plasma membrane fraction and beta actin was used as a marker for cytosolic fraction. **(D)** Wild type and CD36 null murine peritoneal macrophages were incubated with myeloperoxidase (MPO)-modified LDL (oxLDL, 50 μg/ml) for indicated times. The lysates were analyzed by western blot for phosphorylated vimentin (Ser72). **(E)** Macrophages were treated with oxLDL (50 μg/ml) for 10 min. Cytosolic fraction is divided based on the solubility in triton X-100. Western blot for vimentin was done. GAPDH was used as a marker for soluble fraction and flotilin-1 was used as a marker for insoluble fraction. ***p < 0.001. The graph shows mean ± SEM for triplicated determinants of the experiments.

The major regulatory mechanism of vimentin is site-specific phosphorylation (18). We found that oxLDL induced phosphorylation of serine72 (ser72) in a dose-dependent manner (Figures 4A,B). OxLDL induced more vimentin phosphorylation (Ser72) than native LDL (Supplementary Figure 1). However, serine38 of vimentin was not affected by oxLDL (Figure 4A). Subcellular fractionation study showed that phosphorylated vimentin was increased in response to oxLDL both in the cytosolic and the membrane fractions of macrophages (Figure 4C). Vimentin phosphorylation (Ser72) by oxLDL was dependent on CD36. Western blot showed that oxLDL induced vimentin phosphorylation (Ser72) in macrophages from wild type mice while vimentin phosphorylation (Ser72) was not induced by oxLDL in macrophages from CD36 null mice (Figure 4D).

Vimentin phosphorylation at Ser72 is known to impair the assembly of vimentin intermediate filaments during mitosis in fibroblasts (18). To demonstrate the effect of vimentin phosphorylation (Ser72) in macrophages, we separated macrophage lysates into soluble/insoluble fractions based on solubility in triton X-100. In the soluble fraction which contains monomeric vimentin, vimentin was increased after the oxLDL treatment. In accordance, the level of vimentin in insoluble, filamentous fraction was decreased after the oxLDL treatment (Figure 4E).

### Protein Kinase A Mediates Vimentin Phosphorylation (Ser72) Induced by Oxidized LDL

**FIGURE 5:**
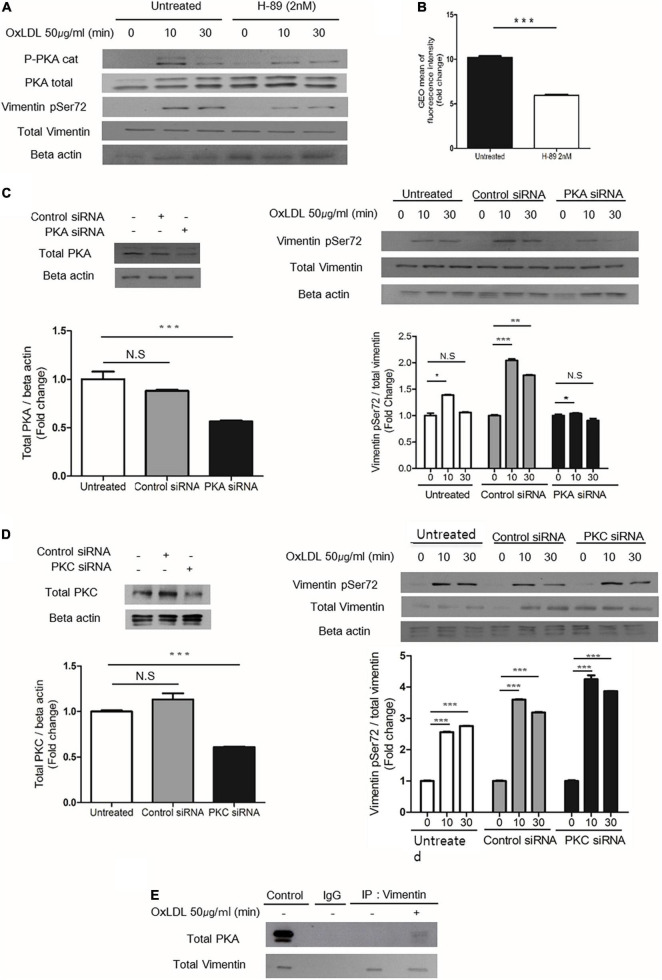
PKA mediates oxLDL-induced vimentin (Ser72) phosphorylation. **(A)** Wild type murine peritoneal macrophages pretreated with H-89(2 nM), a specific PKA inhibitor were incubated with oxLDL (50 μg/ml) for indicated times. The lysates were analyzed by immunoblot for phosphorylated vimentin (Ser72) and total vimentin. Beta actin was used for internal control. **(B)** Flow cytometry data. BMDMs were pretreated with or without H-89 (2 nM) and then exposed to DiI- oxLDL (50 μg/ml) for 5 min. The fluorescence intensities of these cells were measured. **(C)** Left, Western blot analysis for PKA was performed using cell lysates of wild type murine BMDM transfected with control siRNA or siRNA against PKA. Cells were lysed 24 h after the siRNA treatment and analyzed by immunoblot for PKA. Right: Wild type murine BMDMs were transfected with control siRNA or siRNA against PKA and incubated with oxLDL (50 μg/ml) for indicated times and analyzed by western blot. **(D)** Left: Western blot analysis for PKC was performed using cell lysates of wild type murine BMDM transfected with control siRNA or siRNA against PKC. Cells were lysed 24 h after the siRNA treatment and analyzed by immunoblotting to confirm the suppression of PKC expression. Right: Wild type murine BMDMs were transfected with control siRNA or siRNA against PKC and incubated with oxLDL (50 μg/ml) for indicated times and analyzed by western blot. **(E)** *Vim* ^+/+^ murine peritoneal macrophages were treated with or without oxLDL (50 μg/ml) for 10 min and immunoprecipitated with anti-vimentin antibody. The precipitants were analyzed by western blot for PKA. *p < 0.05, **p < 0.01, ***p < 0.001.

To identify the specific kinase that phosphorylates vimentin in response to oxLDL, we blocked various kinases using inhibitors as staurosporine, IPA-3, LY294002 and H-89. We found that H-89, a specific inhibitor for Protein Kinase A (PKA) (40), reduced oxLDL-induced vimentin phosphorylation (Ser72). We also found that oxLDL induced phosphorylation of PKA (PKA α/β/γ-c, Thr198) (Figure 5A). In accordance, H-89-treated wild type macrophages internalized 50% less DiI-oxLDL than H-89-untreated macrophages (Figure 5B).

BMDMs transfected with PKA-specific siRNA showed less oxLDL-induced phosphorylation of vimentin (Ser72) than control siRNA-transfected cells (Figure 5C). However, BMDMs transfected with siRNA against protein kinase C (PKC) which is also known to phosphorylate vimentin (41) did not show changes in oxLDL-induced vimentin phosphorylation (Ser72) (Figure 5D). To see if PKA is physically associated with vimentin, we performed immunoprecipitaion using anti-vimentin antibody and immunoblotting with anti-PKA antibody. The result showed that oxLDL treatment for 5 min induced association between PKA and vimentin (Figure 5E).

These data demonstrated that PKA is activated by oxLDL and mediates vimentin phosphorylation (Ser72).

### Vimentin (Ser72) Phosphorylation Induced by Oxidized LDL Regulates Membrane Localization of CD36 and Is Necessary for Uptake of Oxidized LDL

**FIGURE 6:**
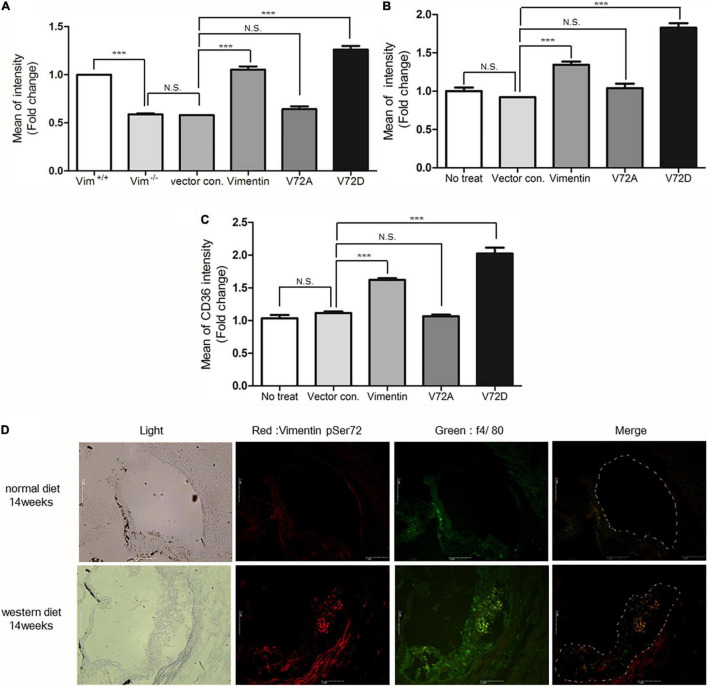
Vimentin (Ser72) phosphorylation induced by oxLDL is required for the uptake of oxLDL. **(A)** Flow cytometry. Fluorescence intensities of diI-oxLDL-treated *Vim*^−/–^ BMDM. Cells were transfected with vectors for vimentin or vimentin mutants. **(B)** Flow cytometry. Geometric mean fluorescence intensities of diI-oxLDL-treated SW13 cells. Cells were transfected with vectors for vimentin or vimentin mutants. **(C)** Measurement of membrane CD36. *Vim*^−/–^ BMDM were transfected with vimentin or mutant vimentin vectors. Stabilized macrophages were stained with APC conjugated anti-CD36 antibody and analyzed using flow cytometry. **(D)** The representative immunohistochemistry images of aortic sinuses *of Ldlr*^−/–^ mice fed a normal diet for 14 weeks or western diet for 14 weeks. Both samples were stained with antibody against phosphorylated vimentin (Ser72) (Dylight 594; Red) and antibody against f4/80 (Alexa 488; Green), a macrophage marker. Scale bars represent 1 μm. N.S. not significant, ***p < 0.001.

To evaluate the role of phosphorylated vimentin (Ser72), we generated Ser72-to-Asp phospho-mimicking mutant (V72D) and Ser72-to-Ala (V72A) non-phospho-mimicking mutant using site-directed mutagenesis. *Vim*^−/–^ BMDM showed 40% less DiI-oxLDL uptake than *Vim*^+/+^ BMDM and *Vim*^−/–^ BMDM that restored vimentin expression showed increased uptake of oxLDL. *Vim*^−/–^ BMDM transfected with V72D showed 1.3 and 2.0-fold higher rates of DiI-oxLDL uptake than vimentin or V72A-transfected macrophages, respectively (Figure 6A).

The site-directed mutagenesis experiments were repeated using SW13 cells, renal epithelial cell line not expressing vimentin. SW13 cells transfected with phospho-mimicking mutant (V72D) showed 1.4 fold increased uptake of DiI-oxLDL compared with SW13 cells with wild type vimentin (Figure 6B).

Since *Vim*^−/–^ mouse macrophages showed less CD36 in plasma membrane than *Vim*^+/+^ mouse macrophages, we hypothesized that vimentin phosphorylation may influence CD36 trafficking from cytosol to membrane. To prove the hypothesis, we stained plasma membrane CD36 with fluorescently labeled anti-CD36 antibody and performed flow cytometry to measure the fluorescence intensities that represent the amount of CD36 localized in plasma membrane. CD36 localized in plasma membrane was 2.0 fold more in the macrophages with V72D compared with macrophages with V72A (Figure 6C). These data suggest that vimentin (Ser72) phosphorylation promotes translocation of CD36 to plasma membrane.

To evaluate if atherosclerotic arterial lesion has higher levels of phosphorylated vimentin (Ser72) in macrophages, we performed immunostaining of cross-sections of the aortic sinuses of *Ldlr* ^–/–^ mice fed a normal chow diet and *Ldlr* ^–/–^ mice fed a western diet for 14 weeks. The result showed that macrophages in the atherosclerotic plaque of the western diet-fed mice had higher levels of phosphorylated vimentin (Ser72) compared with macrophages in the aortic sinuses of the chow-diet fed mice (Figure 6D).

### Deletion of Vimentin in Macrophages Reduces Atherosclerotic Lesion Development in *Ldlr*^−/–^ Mice

**FIGURE 7:**
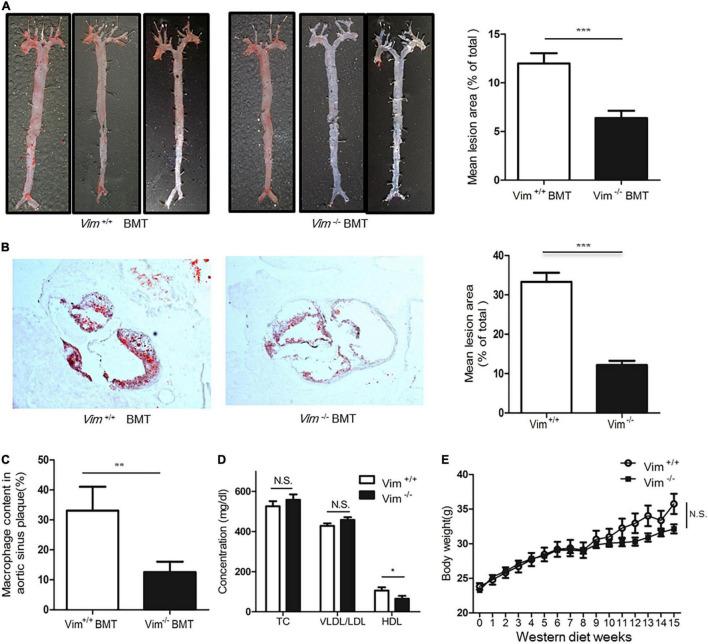
Loss of vimentin in macrophages reduces atherosclerotic lesion in *Ldlr*^−/–^ mice. Lethally irradiated *Ldlr*^−/–^ mice were reconstituted with bone marrow from *Vim*^+/+^ mice (*n* = 11) and *Vim*^−/–^ mice (*n* = 10). After 4 weeks, reconstituted *Ldlr*^−/–^ mice were fed a western diet for 15 weeks. **(A)** Left: Representative en face aortae stained with ORO of *Ldlr*^−/–^ mice transplanted with *Vim*^+/+^ and *Vim*^–/–^ bone marrow. Right: Quantification of percent lesion area in the en face preparations of aortae. Bars indicate mean ± SEM; mice with *Vim* ^+/+^ bone marrow (*n* = 11) and mice with *Vim*^−/–^ bone marrow (*n* = 10). **(B)** Left: Representative cross sections of aortic sinuses stained with ORO, of mice transplanted with *Vim*^+/+^ and *Vim*^–/–^ bone marrow. Right: Quantification of ORO (+) lesion area percentage of the cross sections of the aortic sinuses of mice with *Vim* ^+/+^ bone marrow and *Vim*^−/–^ bone marrow. Bars indicate mean ± SEM; *Ldlr*^−/–^ mice with *Vim* ^+/+^ bone marrow (*n* = 11) and *Vim*^−/–^ bone marrow (*n* = 10). **(C)** Histomorphometric measurements of staining with F4/80, a specific marker of macrophages are shown in bar graphs. Bars indicate mean ± SEM; *Ldlr*^−/–^ mice with *Vim* ^+/+^ bone marrow (*n* = 11) and *Vim*^−/–^ bone marrow (*n* = 10). **(D)** Blood lipid profile of the *Ldlr*^−/–^ mice with *Vim* ^+/+^ bone marrow (*n* = 11) and *Vim*^−/–^ bone marrow (*n* = 10) in **(A)**. TC; Total cholesterol, Bars indicate mean ± SD. **(E)** Body weight changes of the *Ldlr*^−/–^ mice with *Vim* ^+/+^ bone marrow (*n* = 11) and *Vim*^−/–^ bone marrow (*n* = 10) during the western diet period. Bars indicate mean ± SEM. *p < 0.05, **p < 0.01, ***p < 0.001.

To investigate whether deletion of vimentin in macrophages affects the formation of atherosclerotic lesions, we transplanted bone marrow from *Vim*^+/+^ and *Vim*^−/–^ mice to *Ldlr*^−/–^ mice and placed these mice on a western diet for 15 weeks. En face aortae with ORO staining showed that *Ldlr*^−/–^ mice with *Vim*^−/–^ bone marrow had less atherosclerotic lesion than *Ldlr*^−/–^ mice with *Vim*^+/+^ bone marrow (13.13 4.42% vs. 7.464 1.95%, *p*-value = 0.0019, Figure 7A). The cross sections of the aortic sinuses stained with ORO also showed that atherosclerotic plaques are smaller in *Ldlr*^−/–^ mice with *Vim*^−/–^ bone marrow than in *Ldlr*^−/–^ mice with *Vim*^+/+^ bone marrow (Figure 7B). Macrophage counts obtained from F4/80 staining of the cross sections showed that *Ldlr*^−/–^ mice with *Vim*^−/–^ bone marrow had 60% less macrophage number in the atherosclerotic plaque area compared to *Ldlr*^−/–^ mice with *Vim*^+/+^ bone marrow (Figure 7C).

Blood VLDL/LDL and total cholesterol concentrations of these two groups of mice were not different, however, the HDL concentrations of *Ldlr*^−/–^ mice with *Vim*^−/–^ bone marrow were 20% less than *Ldlr*^−/–^ mice with *Vim*^+/+^ bone marrow (Figure 7D). The body weight of *Ldlr*^−/–^ mice with *Vim*^−/–^ bone marrow and *Ldlr*^–/–^ mice with *Vim*^+/+^ bone marrow were not significantly different (Figure 7E).

These data suggest that macrophage vimentin contributes to the development of atherosclerosis.

We generated *Apoe***^–^**^/^**^–^** *Vim* **^–^**^/^**^–^** mice and fed a high fat diet for 15 weeks. Atherosclerotic lesions of aortae were analyzed by ORO staining of the en face bloc. *Apoe***^–^**^/^**^–^** *Vim* **^–^**^/^**^–^** mice showed 57% less atherosclerotic lesions in the aortae than *Apoe***^–^**^/^**^–^** *Vim*^+/+^mice. The cross sections of the aortic sinuses stained with ORO also showed that atherosclerotic plaques are smaller in *Apoe***^–^**^/^**^–^** *Vim* **^–^**^/^**^–^** mice than *Apoe***^–^**^/^**^–^** *Vim*^+/+^mice (Supplementary Figure 2).

We concluded that vimentin promotes atherosclerosis in hypercholesterolemic mice.

## Discussion

Vimentin, the most abundant intermediate filament protein in mesenchymal cells, is well known as a structural protein that supports distribution of intracellular organelles. Vimentin attaches to endoplasmic reticulum, mitochondria and nucleus (41). Several groups made *Vim*^–/–^ transgenic mice to evaluate the role of vimentin *in vivo*, but *Vim*^–/–^ mice did not show a specific phenotype, which has caused limited number of studies for the role of vimentin (42). Vimentin is used as a marker of mesenchymal cells and thus used to assess epithelial-mesenchymal transition (EMT) in malignancy. Recent studies have found several physiological roles of vimentin related to endocytosis. Fay and Panté reported that vimentin is required for parvovirus infection in which vimentin mediates endosomal trafficking of viral particle (13). Sarria et al.’s found that vimentin*-*lacking adrenal cells and normal adrenal cells have the same ability to internalize LDLs but have different abilities in storing LDLs. They suggested that vimentin plays a role in transport of LDL-derived cholesterol from lysosomes to mitochondria, the site for esterification of cholesterol in adrenal cells (43). Heid et al.’s found that vimentin is one of the lipid droplet components in adipocytes based on the proteomic analyses of the components of lipid droplets in adipocytes (44).

We demonstrated that *Vim*^−/–^ mouse peritoneal macrophages uptake less oxLDL than *Vim*^+/+^ mouse peritoneal macrophages, which was proven by DiI-oxLDL uptake assay and oil-red O staining of macrophages incubated with oxLDL (Figure 1).

To clarify which stage of lipid uptake was affected by vimentin, we used U18666A, a specific inhibitor for NPC-1 (Niemann-Pick disease, type C1) (45). NPC-1 is a membrane protein that has a role in intracellular transport of cholesterol from late endosomes to post-lysosomal destinations (46). Blocking NPC-1 protein halts the movement of cholesterol after the late endosome formation. Therefore, we could assess the oxLDL uptake or the early endosome formation distinguished from late endosome. The difference in oxLDL uptake between *Vim* ^+/+^ and *Vim* ^–/–^ mouse peritoneal macrophages was increased after blocking NPC-1. Therefore, we concluded that vimentin functions in foam cell formation via mediating uptake of oxLDL in macrophages.

We found that CD36 localized in plasma membrane was 50% less in *Vim*^−/–^ macrophages than in *Vim* ^+/+^ macrophage. Despite less uptake of oxLDL in *Vim* ^–/–^ macrophages, there was no difference in the amount of CD36 proteins in whole cell lysates between *Vim* ^+/+^ and *Vim* ^–/–^ macrophages. We showed that vimentin–CD36 co-localization was increased by oxLDL in macrophages using various techniques including immunoprecipitaion, immunostaining, and sucrose gradient early endosomal fraction assay.

Vimentin has a central alpha-helical domain and capped on each end by non-helical domain. Two vimentin monomers form a coil-coil dimer, which forms the basis for the filamentous vimentin and this process is known to be regulated by phosphorylation of vimentin. We found that oxLDL/CD36 interaction induced vimentin (Ser72) phosphorylation via PKA (Figure 1). We observed that phospho-mimetic of vimentin (Ser72) increased oxLDL uptake and membrane CD36 localization. In summary, our studies revealed that oxLDL/CD36 interaction induces phosphorylation of vimentin (Ser72) and phospho-vimentin (Ser72) promotes CD36 trafficking to the plasma membrane.

CD36 mediates various cellular activities including fatty acid transport, engulfing virus-infected cells and oxLDL uptake (47). Macrophage CD36 expression is known to be promoted by its ligand, oxLDL, which is called “eat me signal.” CD36 transcription is induced by oxLDL via activation of peroxisome proliferator-activated receptor γ (PPARγ) (48). Both 9- hydroxyoctadecadienoic acid (9-HODE) and 13-HODE in oxLDL serve as endogenous PPAR γ ligands. To reduce foam cell formation and treat atherosclerosis, there have been various efforts to reduce CD36 expression. Those includes usage of drugs such as α-tocopherol and tamoxifen. However, no drugs have achieved enough efficacy in clinical settings (49). Therefore, modulating functions of vimentin may be one way to inhibit macrophage foam cell formation. Regarding our finding, oxLDL increases uptake of itself in macrophages via two ways; one is increase of CD36 transcription and the other is increase of membrane trafficking of CD36.

Recently, Haversen et al. reported that vimentin deficiency increased membrane-localized CD36, resulting in increased uptake of oxLDL (29), which is an opposite observation to our finding. However, animal experiment using *Vim*^–/–^ bone marrow transplantation in their study showed the same finding to ours that *LDLR*^–/–^ mice reconstituted with *Vim*^–/–^ bone marrow had less atherosclerotic lesions than *LDLR*^–/–^ mice with *Vim*^+/+^ bone marrow. Contrary findings in cellular experiments may have been caused by different experimental settings. Peritoneal macrophages and BMDMs are known to be phenotypically distinct and differ in expression of M1/M2 markers and lipid metabolism genes (50). Differences in methods to achieve BMDMs could contribute to different responses of the cells. We differentiated murine bone marrow myeloid stem cells into BMDMs by culturing in DMEM supplemented with 10% L929 supernatant and 10% fetal bovine serum (FBS) for 7 days. Harversen et al. used high-glucose DMEM supplemented with 10% whole supernatant of cell line CMG14-12 as a source of mouse M-CSF.

To clarify the differences in baseline CD36 expression between BMDM and peritoneal macrophages, we measured cell surface CD36 in both types of cells. FACS analysis using anti-CD36 antibody showed that 3.02 times higher intensity was measured in peritoneal macrophages (Supplementary Figure 3). Peritoneal macrophages released more TNF-α in response to oxLDL and MCP-1 than BMDM (Supplementary Figure 4).

Although baseline expressions of CD36 and other molecules are different between BMDM and peritoneal macrophages, in our current study, we attained consistent results that vimentin deficiency reduces expression of cell surface CD36 and thus reduces uptake of oxLDL in both BMDM and peritoneal macrophags (Figures 2D,E). We also conducted site-specific mutagenesis study using BMDM (Figure 6). As in the experiment using peritoneal macrophages from *Vim*^–/–^ and *Vim*^+/+^ mice (Figure 1), BMDM from *Vim*^–/–^ mice showed 40% less oxLDL uptake than BMDM from *Vim*^+/+^ mice or *Vim*^–/–^ BMDM that restored vimentin expression (Figure 6A) and also showed less expression of plasma membrane CD36 (Figure 6C).

Another report revealing that the inhibitory effect of tetrahydroxystilbene glucoside on macrophage foam cell formation is driven by reduction of vimentin (51) also supports our observation. In a different study of ours, we also showed that membrane-localized CD36 in *Vim*^–/–^ adipocytes was 41∼ 58% less than in control *Vim*^+/+^ adipocytes and thus fatty acid uptake of *Vim*^–/–^ adipocytes was 27% less than *Vim*^+/+^ adipocytes (52).

We measured LDLR expression in peritoneal macrophages from *Vim*^+/+^ and *Vim* **^–^**^/^**^–^** mice. The expression of LDLR was 1.37 fold higher in *Vim* **^–^**^/^**^–^** macrophages (Supplementary Figure 5).

Differences in oxLDL preparations could attribute to the disparity among *in vitro* studies. We generated CuSO_4_-oxLDL and measured oxidation degree of the CuSO_4_-oxLDL by TBARS assay (thiobarbituric acid reactive substance assay). Our oxLDL was 8–10 nmol TBARS/mg protein MDA equivalents which could be classified as mildly oxidized LDL (Supplementary Figure 6). It has been reported that mildly oxidized LDL is chemically different from unmodified LDL and has more affinity to CD36 than the other scavenger receptors including scavenger receptor-A (53). We also used myeloperoxidase (MPO)-modified LDL in our assay to evaluate effect of CD36 in oxLDL-induced vimentin (Ser72) phosphorylation (Figure 4D). MPO is physiological oxidizing reagent in the human body and thus MPO-modified LDL should be closer to the endogenously oxidized LDL. We achieved consistent results from the assays using CuSO_4_-oxLDL and MPO-modified LDL.

A recent study by Wang et al. revealed that palmitoylation of CD36 by DHHC4 and DHHC5 is required for its plasma membrane localization and fatty acid uptake activity (54). It is possible that phospho-vimentin may affect palmitoylation of CD36 via modulating activities of palmitoyl-acyltransferase like DHHC4 and DHHC5. The question we should solve is whether CD36 increase in plasma membrane is derived from achieving stability of membrane-localized CD36 or it is from increased dynamic trafficking (fast receptor recycling via increased dynamic assembly and disassembly of vimentin intermediate filament). This question is on our venue of on-going research.

In summary, our current study demonstrated that vimentin-deficient macrophages uptake less oxLDL via decreased membrane localization of CD36 and thus CD36-deficiency in macrophages reduces development of atherosclerosis. We suggest underlying mechanisms that oxLDL/CD36 interaction-induced vimentin (Ser72) phosphorylation leads to disassembly of vimentin filament and vimentin mediates membrane trafficking of CD36 and receptor-mediated endocytosis of CD36. Our study suggests a new therapeutic strategy for the treatment of atherosclerosis by revealing a new function of vimentin in macrophages. It may also suggest ways to regulate CD36 trafficking and modulate functions of CD36 in various biological activities including fatty acid transport in adipocytes, immunity, and anti-angiogenesis.

## Data Availability Statement

The original contributions presented in the study are included in the article/Supplementary Material, further inquiries can be directed to the corresponding author/s.

## Ethics Statement

The animal study was reviewed and approved by Institutional Animal Care and Use Committee (IACUC) of Ewha Womans University College of Medicine.

## Author Contributions

SK designed and performed the work. S-JJ, J-HP, and WC contributed to the acquisition and analysis of the work. Y-HA and Y-HC designed the work. GO and RS contributed to interpretation of the work. YP designed and analyzed the work. All authors contributed to the article and approved the submitted version.

## Conflict of Interest

The authors declare that the research was conducted in the absence of any commercial or financial relationships that could be construed as a potential conflict of interest.

## Publisher’s Note

All claims expressed in this article are solely those of the authors and do not necessarily represent those of their affiliated organizations, or those of the publisher, the editors and the reviewers. Any product that may be evaluated in this article, or claim that may be made by its manufacturer, is not guaranteed or endorsed by the publisher.
