# Supplementary material for: Plasma Membrane Localization of CD36 Requires Vimentin Phosphorylation; A Mechanism by Which Macrophage Vimentin Promotes Atherosclerosis
Source: Front Cardiovasc Med. 2022 May 18;9:792717. doi: 10.3389/fcvm.2022.792717 (PMC9152264; doi:10.3389/fcvm.2022.792717)
Supplement: Supplementary file 1 [file Data_Sheet_1.PDF]

Supplementary Figures

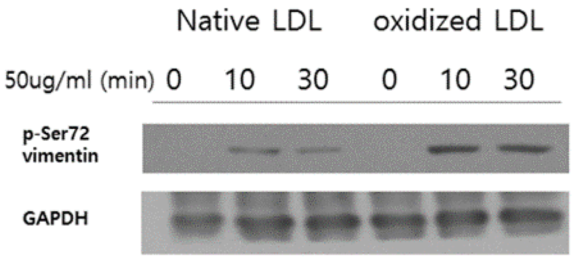

Supplementary Fig.1 Vimentin (Ser72) phosphorylation by oxLDL depends on oxidation degree of the LDL

Wild type macrophages were treated with $CuSO_4$-oxLDL and native LDL. The cell lysates were applied to the western blot for p-Vimentin (Ser72). Oxidation degree of the native LDL was 7 nmol_TBARS/mg protein MDA equivalents while oxLDL showed 10 nmol TBARS/mg protein MDA equivalents as in Supplementary Fig.4. (LDL might contain endogenous oxLDL and thus minimally oxidized in this assay.)

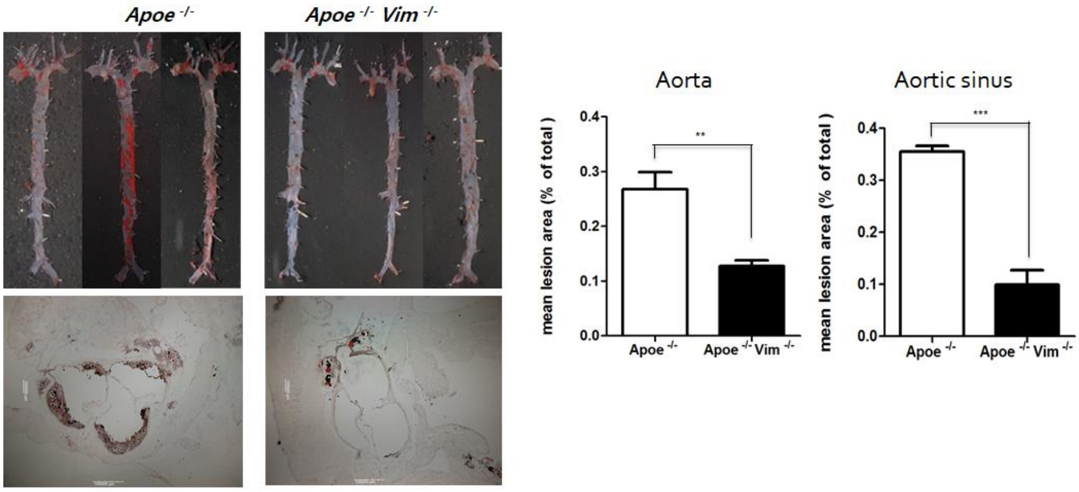

Supplementary Fig.2 Vimentin deficiency reduces atherosclerosis in *Apoe$^{-/-}$Vim$^{-/-}$* mice.

Apoe$^{-/-}$Vim$^{-/-}$ mice and *Apoe$^{-/-}$Vim$^{+/+}$* mice were fed a western diet for 15 weeks. En face aortae were stained with oil Red-O (ORO) and atherosclerotic lesions stained with ORO were counted. (Left upper panels).

Aortic sinus sections were stained with ORO (Left lower panels). Quantification of the mean ORO (+) area (Right graphs). $^{**}p < 0.01$, $^{***}p < 0.001$

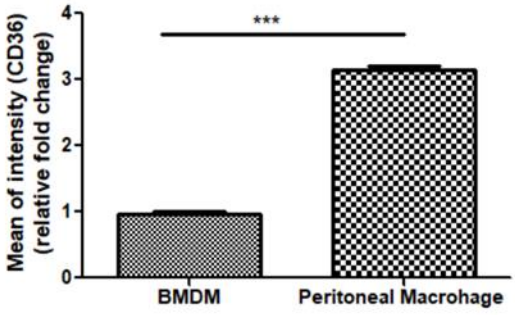

Supplementary Fig.3 Cell surface CD36 expression in murine bone marrow derived macrophages and peritoneal macrophages

Bone marrow derived macrophages (BMDM) and peritoneal macrophages from wild type were stained with fluorescent anti-CD36 antibody. Fluorescence intensities representing cell surface CD36 expression were measured by flow cytometry. ****p* < 0.001

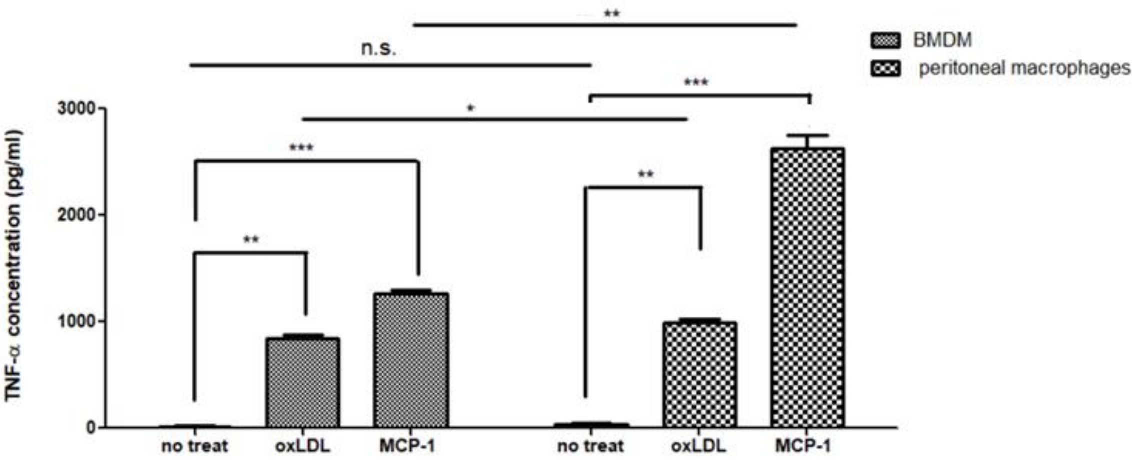

Supplementary Fig.4 TNF-α production of PEMs and BMDMs in response to oxLDL and MCP-1

Wild type murine peritoneal macrophages and bone marrow derived macrophages were incubated with oxLDL (50 μg/ml) and MCP-1 (25ng/ml) for 6 hours. The media were analyzed by ELISA. (*: p <0.05, **: p<0.01, *** p<0.001. The graph shows mean ± SEM for triplicated determinants of 3 seperate experiments.)

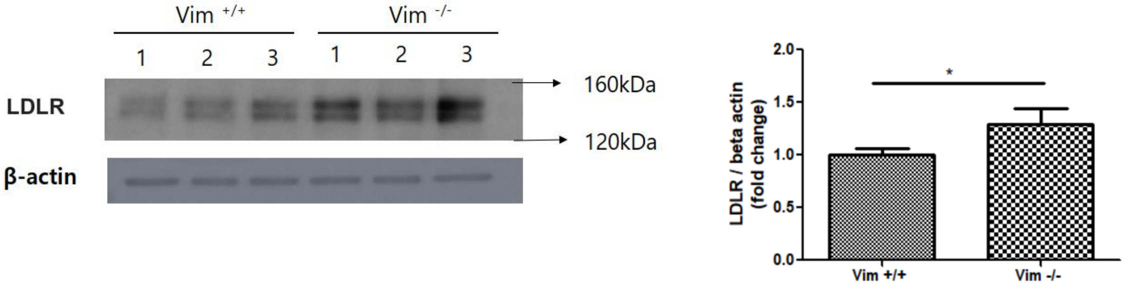

Supplementary Fig.5 LDLR expression in *Vim* $^{+/+}$ and *Vim* $^{-/-}$ macrophages

Peritoneal macrophages from *Vim* $^{+/+}$ and *Vim* $^{-/-}$ mice were lysed and applied to western blot for LDLR. The numbers of the lanes indicate macrophage lysates from different mice. *$p<0.1$

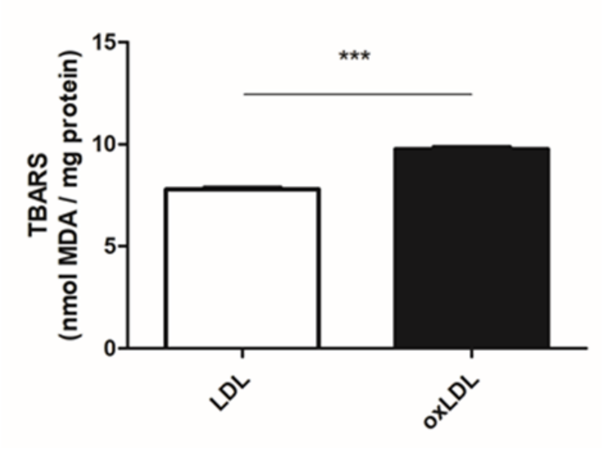

Supplementary Fig.6 TBARS assay to measure oxidation degree

LDL was obtained from human plasma via density gradient ultracentrifugation. OxLDL was generated by dialysis of LDL with 5μM $CuSO_4$ in PBS for 6 hours at 37°C. To terminate oxidation of LDL, LDL was dialyzed with 100μM EDTA in PBS. We measured oxidation degree of oxLDL and LDL (processed without $CuSO_4$) by TBARS assay. (LDL might contain endogenous oxLDL and thus minimally oxidized in this assay.)
